# Correction to: Explicit upper bound for the average number of divisors of irreducible quadratic polynomials

**DOI:** 10.1007/s00605-018-1177-8

**Published:** 2018-03-24

**Authors:** Kostadinka Lapkova

**Affiliations:** Institute of Analysis and Number Theory, Graz University of Technology, Kopernikusgasse 24/II, 8010 Graz, Austria

**Keywords:** Number of divisors, Quadratic polynomial, Character sums, Primary 11N56, Secondary 11D09

## Correction to: Monatsh Math 10.1007/s00605-017-1061-y

Let $\tau (n)$ denote the number of positive divisors of the integer *n*. In [3], we provided an explicit upper bound for the sum $\sum _{n=1}^N\tau \left( n^2+2bn+c\right)$ under certain conditions on the discriminant, and we gave an application for the maximal possible number of $D(-1)$-quadruples.

The aim of this addendum is to announce improvements in the results from [3] . We start with sharpening of Theorem 2 [3].

### Theorem 2A

Let $f(n)=n^2+2bn+c$ for integers *b* and *c*, such that the discriminant $\delta :=b^2-c$ is nonzero and square-free, and $\delta \not \equiv 1\pmod 4$. Assume also that for $n\ge 1$ the function *f*(*n*) is nonnegative. Then for any $N\ge 1$ satisfying $f(N)\ge f(1)$, and $X:=\sqrt{f(N)}$, we have the inequality

$$\begin{aligned} \sum _{n=1}^N \tau (n^2+2bn+c)&\le \frac{2}{\zeta (2)}L(1,\chi ) N\log X \\&\quad + \left( 2.332L(1,\chi )+\frac{4M_\delta }{\zeta (2)}\right) N+\frac{2M_\delta }{\zeta (2)} X\\&\quad +4\sqrt{3}\left( 1-\frac{1}{\zeta (2)}\right) M_\delta \frac{N}{\sqrt{X}}\\&\quad +2\sqrt{3}\left( 1-\frac{1}{\zeta (2)}\right) M_\delta \sqrt{X} \end{aligned}$$

where $\chi (n)=\left( \frac{4\delta }{n}\right)$ for the Kronecker symbol $\left( \frac{.}{.}\right)$ and

$$\begin{aligned} M_\delta =\left\{ \begin{array}{ll} \frac{4}{\pi ^2}\delta ^{1/2}\log {4\delta }+1.8934\delta ^{1/2}+1.668, &{}\quad \mathrm{if }\,\, \delta >0;\\ &{} \\ \frac{1}{\pi }|\delta |^{1/2}\log {4|\delta |}+1.6408|\delta |^{1/2}+ 1.0285,&{}\quad \mathrm{if }\,\, \delta <0. \end{array}\right. \end{aligned}$$

In the case of the polynomial $f(n)=n^2+1$, we can give an improvement to Corollary 3 from [3].

### Corollary 3A

For any integer $N\ge 1$, we have

$$\begin{aligned} \sum _{n\le N}\tau (n^2+1)<\frac{3}{\pi }N\log N +3.0475 N+1.3586 \sqrt{N}. \end{aligned}$$

Just as in [2, 3], we have an application of the latter inequality in estimating the maximal possible number of $D(-1)$-quadruples, whereas it is conjectured there are none. We can reduce this number from $4.7\cdot 10^{58}$ in [2] and $3.713\cdot 10^{58}$ in [3] to the following bound.

### Corollary 4A

There are at most $3.677\cdot 10^{58}$ $D(-1)$-quadruples.

The improvements announced above are achieved by using more powerful explicit estimates than the ones used in [3]. More precisely, the results are obtained when instead of Lemma 2 and Lemma 3 from [3] we plug in the proof the following stronger results.

### Lemma 2A

For any integer $N\ge 1$ we have

$$\begin{aligned} \sum _{n\le N}\mu ^2(n)=\frac{N}{\zeta (2)}+E_1(N), \end{aligned}$$

where $$|E_1(N)|\le \sqrt{3}\left( 1-\frac{1}{\zeta (2)}\right) \sqrt{N}<0.6793\sqrt{N}$$.

### Proof

This is inequality (10) from Moser and MacLeod [4]. $\square$

The following numerically explicit Pólya–Vinogradov inequality is essentially proven by Frolenkov and Soundararajan [1], though it was not formulated explicitly. It supersedes the main result of Pomerance [5], which was formulated as Lemma 3 in [3].

### Lemma 3A

Let

$$\begin{aligned} M_\chi :=\max _{L,P}\left| \sum _{n=L}^P \chi (n)\right| \end{aligned}$$

for a primitive character $\chi$ to the modulus $q>1$. Then

$$\begin{aligned} M_\chi \le \left\{ \begin{array}{ll} \frac{2}{\pi ^2}q^{1/2}\log {q}+0.9467q^{1/2} +1.668\,, &{} \chi \,\, \mathrm{even};\\ &{} \\ \frac{1}{2\pi }q^{1/2}\log {q}+0.8204q^{1/2}+1.0285,&{}\chi \,\, \mathrm{odd}. \end{array}\right. \end{aligned}$$

### Proof

Both inequalities for $M_\chi$ are shown to hold by Frolenkov and Soundararajan in the course of the proof of their Theorem 2 [1] as long as a certain parameter *L* satisfies $1\le L\le q$ and $L=\left[ \pi ^2/4\sqrt{q}+9.15\right]$ for $\chi$ even, $L=\left[ \pi \sqrt{q}+9.15\right]$ for $\chi$ odd. Thus both inequalities for $M_\chi$ hold when $q>25$.

Then we have a look of the maximal possible values of $M_\chi$ when $q\le 25$ from a data sheet, provided by Leo Goldmakher. It represents the same computations of Bober and Goldmakher used by Pomerance [5]. We see that the right-hand side of the bounds of Frolenkov–Soundararajan for any $q\le 25$ is larger than the maximal value of $M_\chi$ for any primitive Dirichlet character $\chi$ of modulus *q*. This proves the lemma. $\square$
